# RNA Nanotechnology-Mediated Cancer Immunotherapy

**DOI:** 10.7150/thno.35568

**Published:** 2020-01-01

**Authors:** Yao-Xin Lin, Yi Wang, Sara Blake, Mian Yu, Lin Mei, Hao Wang, Jinjun Shi

**Affiliations:** 1 Center for Nanomedicine and Department of Anesthesiology, Brigham and Women's Hospital, Harvard Medical School, Boston, MA 02115, USA; 2 CAS Center for Excellence in Nanoscience, CAS Key Laboratory for Biomedical Effects of Nanomaterials and Nanosafety, National Center for Nanoscience and Technology, Beijing 100190, China; 3 School of Pharmaceutical Sciences (Shenzhen), Sun Yat-sen University, Guangzhou, Guangdong 510006, China; 4 Center of Materials Science and Optoelectronics Engineering, University of Chinese Academy of Sciences, Beijing 100049, China; 5 Tufts University, Medford, MA 02155, USA

**Keywords:** RNA, nanoparticle, immunotherapy, cancer, RNAi, CRISPR

## Abstract

RNA molecules (e.g., siRNA, microRNA, and mRNA) have shown tremendous potential for immunomodulation and cancer immunotherapy. They can activate both innate and adaptive immune system responses by silencing or upregulating immune-relevant genes. In addition, mRNA-based vaccines have recently been actively pursued and tested in cancer patients, as a form of treatment. Meanwhile, various nanomaterials have been developed to enhance RNA delivery to the tumor and immune cells. In this review article, we summarize recent advances in the development of RNA-based therapeutics and their applications in cancer immunotherapy. We also highlight the variety of nanoparticle platforms that have been used for RNA delivery to elicit anti-tumor immune responses. Finally, we provide our perspectives of potential challenges and opportunities of RNA-based nanotherapeutics in clinical translation towards cancer immunotherapy.

## 1. Introduction

Over the past two decades, RNA-based therapeutics, such as messenger RNA (mRNA), microRNA, and small interfering RNA (siRNA), have emerged as highly attractive classes of drugs for the treatment and prevention of numerous diseases (e.g., cancers, genetic disorders, diabetes, inflammatory diseases, and neurodegenerative diseases)^1^-^6^. In comparison to the conventional small molecular drugs or proteins (specifically antibodies), RNA therapeutics can play remarkable regulatory roles in the treatment of targeted cells by either increasing the expression of a given protein or knocking out targeted genes to some varying degree. In addition to these regulatory roles, the fact that they are more convenient and easier to design than protein-based drugs, is what makes RNA therapeutics such an appealing form of treatment to researchers. However, the instability of RNAs themselves and the presence of various physiological barriers that inhibit the delivery and transfection of RNAs are what hinder their clinical application in cancer therapy 7, 8. Moreover, the exogenous RNA is more likely to be cleared by the human body's intrinsic defense systems, e.g., various exonucleases and RNases responsible for RNA degradation, the major organs or tissues (e.g., kidneys and liver), and the innate immune system for RNA clearance 3, 9. To overcome such immunogenic hurdles and make sure safe delivery of these RNA therapeutics to their target sites occurs, nanoparticle-based delivery systems have been explored as potential RNA delivery tools for *in vitro* and *in vivo* applications 10, 11.

Since the clinical success of immune checkpoint blockade (ICB)^12^ and chimeric antigen receptor (CAR) T-cell therapies^13^, ^14^, cancer immunotherapy treatments have drawn increasing interests. In contrast to chemotherapeutic drugs with dose-limited toxicities and potential development of drug-resistance by tumor cells, immunotherapeutics can inhibit the ability of tumor cells to evade termination by the immune system or re-program cancer-associated immune systems, and are thus more specific and able to trigger long-lasting memory anti-tumor responses. Despite these desirable features and research breakthroughs, currently used ICB antibodies and cell-based therapeutics (e.g., CAR-T) in tumor immunotherapy are far from perfect, and it is imperative to pursue new strategies for improving their safety and efficacy 15-17. RNA-based therapeutics have many potential uses in immunomodulation and cancer immunotherapy, such as silencing immune checkpoint genes, activating the innate or adaptive immune system by regulating cytokines expressions, and acting as tumor antigen vaccines^18^, ^19^. The use of RNA-based therapeutics has recently expanded dramatically, and some have been moved to clinical trial studies during the past decade, revealing these genetic materials as excellent candidates for cancer treatment. Meanwhile, the development of various nanoparticle-based platforms, such as liposomes 20, polymeric nanoparticles (NPs)^21^-^26^, and inorganic NPs^27^, ^28^ for efficient delivery of RNAs provides a bright future for RNA-based therapeutics and their applications in cancer immunotherapy.

In this review article, an overview of RNA-based nanotherapeutics and recent advances, including their delivery nanoplatforms and applications in tumor immunotherapy, will be presented. Also, the various nanomaterials that have been used to deliver RNAs to tumor cells or immune cells for the induction of anti-tumor immune responses, will be highlighted. Finally, the current challenges of RNA-based nanotherapeutics will be discussed and the potential clinical value of RNA-based nanotherapeutics in tumor immunotherapy will be highlighted.

## 2. Nanotechnology for delivery of therapeutic RNAs

### 2.1 RNA therapeutics

**Figure 1:**
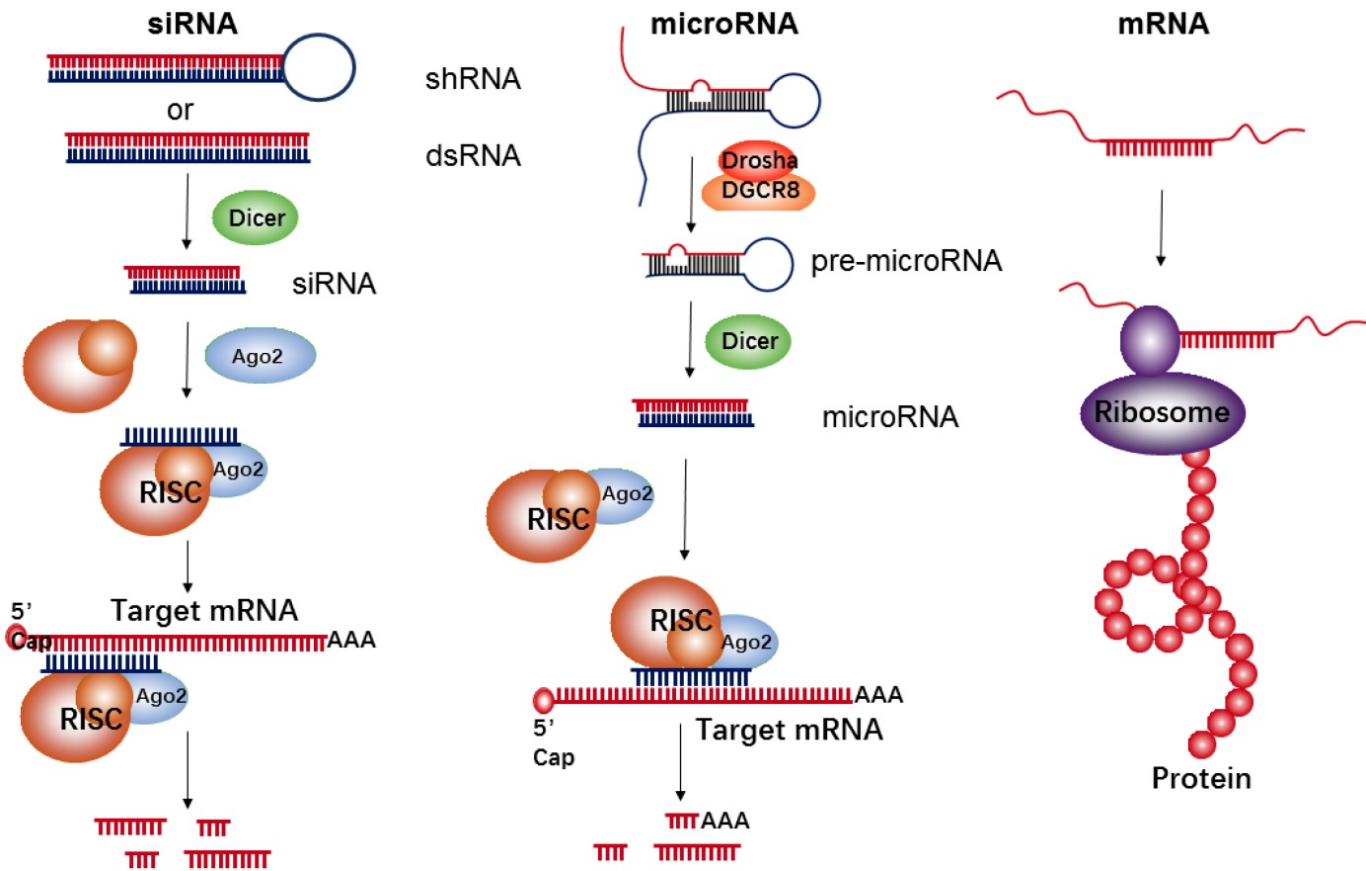
The biological mechanism of siRNA, microRNA, and mRNA for inhibition of target protein expressions or up-regulation of a given protein.

RNA-based therapeutics have demonstrated a wide array of promising applications in the field of cancer treatment. They function as either inhibitors (e.g., siRNA and microRNA) or upregulators (e.g., mRNA) of target protein expression (Figure 1). siRNA is double-stranded in nature and approximately 22 nucleotides in length. Its precursor is initially recognized by Dicer RNase and is then incorporated into the RNA-induced silencing complex (RISC). The siRNA-RISC complex can bind the targeting site of mRNA, and lead to a sequence-specific cleavage by endonuclease Argonaute-2 (AGO2), thus decreasing expressions of a targeted protein 29. MicroRNA is another common short regulatory noncoding RNA, used for blocking target gene expression via binding to target sites in the 3'-untranslated regions (UTR) of protein-coding transcripts 30. Firstly, primary microRNA (pri-microRNA) with a characteristic hairpin structure is recognized and processed by enzymes of Drosha and DGCR8 into ∼70 nt precursor microRNA (pre-microRNA). The resultant pre-microRNA is further cleaved by Dicer RNase, thus resulting in the formation of a mature dsRNA (microRNA). The mature microRNA is finally incorporated into RISC to induce cleavage of targeted mRNA, such as siRNAs, or translational repression, which induces a decrease of targeted proteins. Generally, the target sequences of the microRNA are frequently found in the 3' UTR of mRNA and can often be found within non-coding or intronic regions. Therefore, each microRNA can be capable of targeting hundreds of unique mRNAs and inducing regulation of the transcriptome. However, in comparison to microRNA's multi-mRNA targeting abilities, siRNA has specific binding activity; therefore, each siRNA can only bind one mRNA target.

The goal of mRNA delivery is to upregulate targeted protein expressions like DNA delivery, but in contrast to DNA, mRNA therapeutics have several unique features, such as the absent risk of insertional mutagenesis, more consistent and predictable kinetics of protein expression, and relatively convenient *in vitro* synthesis^31^. Meanwhile, the transfection efficiency with mRNA is higher than that of DNA, especially in immune cells^32^-^34^. Each mRNA has an open reading frame (ORF) that includes two untranslated regions (UTRs) located at the 5' and 3' ends of mRNA, with the purpose of being recognized by the translational machinery (Ribosome). In addition to those UTRs, the mRNA's 5' methyl cap and 3' poly(adenosine) tail are also crucial for efficient translation^35^.

### 2.2 Nanocarriers for RNA delivery

**Figure 2:**
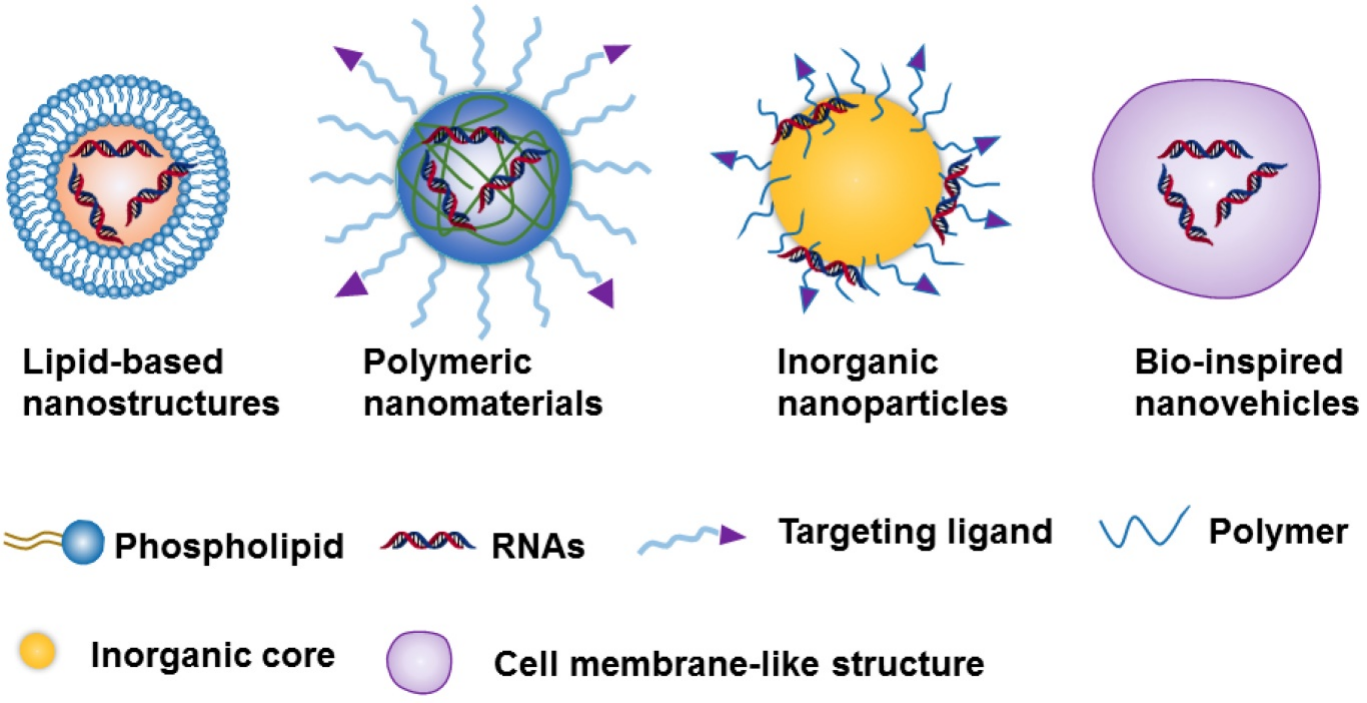
Schematic representation of the 4 nanoparticle-based platforms used in the RNA delivery.

Due to the challenges of naked RNA molecules for *in vivo* applications, i.e., extremely short half-lives (e.g., minutes), poor chemical stability, and easy degradation by nucleases^18^, ^36^, nanotechnology provides a versatile and targeted system for the safe delivery of them 37. The nanoparticle-based delivery systems not only protect RNA molecules from enzymatic degradation and immune system threats, but they also enable RNA accumulation to occur in the tumor site 35, 36. This RNA accumulation is able to occur due to the enhanced permeability and retention effect (EPR), which is a byproduct of the diameter of the NPs, which can range from 10 to 200 nm^35^, ^36^. Currently, the constituents of nanocarriers applied to RNAs delivery can be classified as lipid-based nanosystems 38-42, polymeric nanomaterials 43-45, inorganic nanoparticles 28, 46, 47 ,or Bio-inspired nanovehicles 48, 49 (Figure 2). We will here summarize these nanoparticle-based platforms and further discuss their strengths and potential drawbacks for RNA delivery (Table 1).

#### 2.2.1 Lipid-based nanostructures

Lipid-based nanostructures are one of the most commonly used non-viral delivery systems both in academic studies and clinical trials for chemotherapeutics or genetic drugs. Several classes of lipid-based nanocarriers including liposomes, lipid nanoemulsions, and solid lipid NPs have been used for RNA delivery 50. Generally, cationic lipids could be used as RNAs delivery carriers owing to their positively charged motif that has a strong interaction with negatively charged nucleic acids. As the lipids and phospholipids are the basic units of the cell membrane, lipid-based nanostructures with the similar units have a natural tendency to interact well with the cells membrane and thereby facilitating cellular uptake of RNAs. Meanwhile, the obvious other advantages of lipid-based nanostructures, such as easy preparation, good biocompatibility and biodegradability, have led them to be a promising tool in the delivery of RNA-based therapeutics. However, there are also several issues, including limited stability, easy leakage of payloads, and rapid clearance by the kidneys or liver should be addressed before these lipid-based delivery systems are put to perform at clinical or *in vivo* levels.

#### 2.2.2 Polymer-based nanomaterials

Polymer-based nanomaterials are well-studied systems for RNA delivery 51, which are classified into two major categories: natural or naturally derived polymers, and synthetic polymeric conjugates 43, 44. Natural or naturally derived polymers such as chitosan, which is composed of N-acetyl-d-glucosamine and d-glucosamine, poly-l-lysine, which consists of repeating units of lysine, and atelocollagen, occur in nature and are produced by all living organisms. The advantages of these nanoparticles are good biocompatibility and biodegradability, and low cost of production 52-54. Among the synthetic polymers, poly(dl-lactide-co-glycolide) (PLGA), polyethyleneimine (PEI), polyvinyl alcohol (PVA), polyethylene glycol (PEG), poly-l-lactic acid (PLA), etc., are the most common polymers used in delivery of RNAs due to their high stability, good biocompatibility and biodegradability 55-59. Meanwhile, it is important to note that these synthetic polymers are easy to functionalize with ligand bindings for targeting, and with responsive units that respond to chemical, biological, and physical stimuli for the controllable release of cargoes. However, some synthetic polymers (e.g., PLGA) could not be directly applied in RNA delivery due to no cationic units on them, thus leading to low electrostatic interaction between polymers and RNAs. One method to overcome this problem is modification with various cationic motifs (e.g., PEI) or co-assembly with cationic polymers into nanostructures, it is also noted that the most cationic units or polymers are nondegradable, which may be associated with potential toxicity issues 22.

#### 2.2.3 Inorganic nanoparticles

Recently, various inorganic NPs, such as mesoporous silica nanomaterials (MSNs) 60-62, carbon nanotubes (CNTs) 63, quantum dots (QDs) 64, and metal nanostructures^65^ (e.g., iron oxide and gold NPs), are reported as carriers for delivery of RNAs. These inorganic NPs are synthesized by biodegradable polymers and inorganic particles. Consequently, the properties of these nanoparticles are easy to control, and their advantages include surface modification, good reproducibility, and easy cell uptake. However, the level of degradability of all of the inorganic materials has yet to be determined, and potential toxicity could be a problem 58. As a result, more extensive *in vivo* studies are still needed.

#### 2.2.4 Bio-inspired nanovehicles

In recent studies, some bio-inspired nanovehicles, such as DNA-based nanostructures 66, 67, exosome-mimetic nanovesicles 68, 69, and red cell member-based ghosts 70 have been explored as gene and drug carriers to enhance delivery to targeted cells. The exosome is a bilayer membrane-coated vesicle secreted by cells, whose function is triggering intercellular communication by transferring payload mRNA, miRNA, or proteins from one cell to another. Studies have shown that these exosome nanovesicles exhibit good biodegradability, which can be attributed to their morphology and surface properties which are similar to those of natural cells. Originated from cell membrane proteins, cell membrane-based ghosts are another cell-derived nanovesicles, which possess identical physiochemical properties, such as topological/physiological activities. These cell membrane-based ghosts have a promising future in nanotechnology for a variety of biomedical applications, such as targeted delivery of RNA therapeutics, and tissue regeneration 71. In comparison to other drug delivery systems, the advantages of these bio-inspired nanovehicles are low toxicity, strong targeting abilities, and low immune response induction. However, the high cost of production and vesicle stability should be considered in further clinical applications.

## 3. RNA nanotherapeutics for tumor immunotherapy

Tumor immunotherapy can induce a synergistic killing effect on tumor cells through primarily targeting the immune system rather than the tumor cells themselves. Current immunotherapeutics, including antibodies, proteins, and engineered immune cells (e.g., CAR-T) are promising strategies for cancer treatment and some of them have been successful in clinical applications. However, these therapies still face some critical issues, such as insufficient efficacy (e.g., ICB has shown activity in approximately 15%-25% of patients 72), high costs and side-effect 15, 17. RNA-based agents provide some advantages in immunomodulation, such as high selectivity for silence or increased expression of specific targets and low risk of off-target hitting. Using RNA therapeutics in conjunction with nanomaterials is a valuable strategy to improve the efficacy of immunomodulation for cancer treatments. When used as partners in synergistic combinations, more efficient and personalized results will be reached, enabling the combination to compete with current immunotherapies already on the market.

### 3.1 Nanostructure-mediated siRNA delivery for immunotherapy

Recently, many approaches have demonstrated that silencing the crucial factors of tumor progression in cancer cells or knocking out/down the immunosuppressive genes in cancer 73 and cancer-associated immune cells can effectively induce an anti-tumor immune response 74, 75. Nanomaterials can act as Trojan horses by delivering siRNAs, for regulating the immune response, to cancer or immune cells 76, 77. Here, we summarize the potential siRNA targets in tumor or immune cells, as well as nanostructure delivery systems for siRNA-mediated immunotherapy (Table 2).

### 3.1.1 Tumor cells-targeted siRNA immunenanotherapy

Tumor cells can develop numerous strategies to promote immune system escape and drive the generation of an immunosuppressive microenvironment. For example, tumor cells can effectively induce and recruit distinct immunosuppressive cells, by promoting the secretion of some pro-tumor cytokines and chemokines^107^, such as interleukin-6 (IL-6), IL-10, transforming growth factor-β (TGF-β), vascular endothelial growth factor (VEGF), and C-X-C motif chemokine 5 (CXCL5). Meanwhile, the tumor-infiltrating suppressive cells evade immune surveillance by secreting or expressing a few immunosuppressive molecules that disrupt antigen presentation of dendritic cells (DCs) and suppress proliferation and activation of T cells 108, 109. Additionally, tumor cells could evade immunological eradication by downregulating antigen expression, and upregulating the expression of immune checkpoint proteins (e.g., PD-L1) or “don't eat me” signals (e.g., CD47) 110, 111. CD47 is over-expressed on the cancer cell surface, which enables escape from immune system recognition by labeling the cells with the “self” marker.

**Figure 3:**
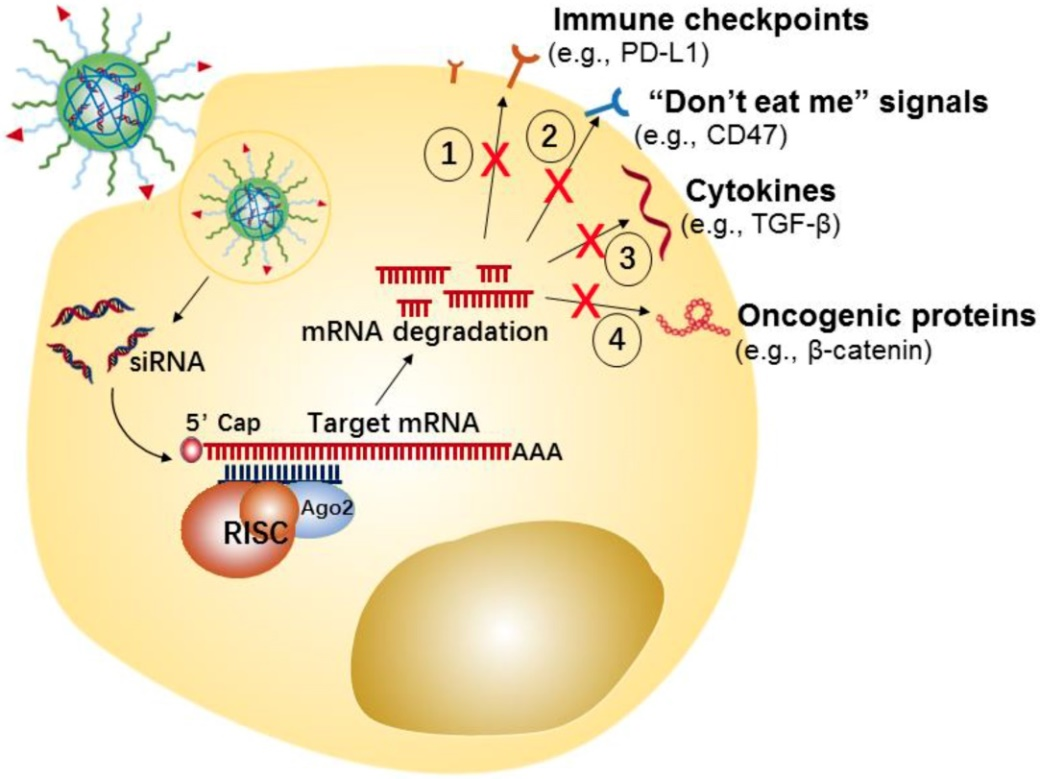
Potential strategies of tumor cells-targeted siRNA nanotherapeutics for cancer immunotherapy.

In the last few years, tumor cell-targeted siRNA nanotherapeutics have been centered on the downregulation of immune checkpoint proteins, “don't eat me” signals, anti-inflammatory cytokines, etc., for inducing anti-tumor immune-responses has been gaining increasing attention (Figure 3). For example, Yang et al. developed a systemic delivery strategy based on HA-coated Lipid NPs for delivery of CD47 siRNA to melanoma cancer cells, which induced an efficient knockdown of CD47 in cancer cells 85. CD47 silence effectively suppressed the tumor growth in a melanoma mice model. Similarly, using siRNA-based nanotherapy for the direct knockdown of immune checkpoints or anti-inflammatory cytokines on tumor cells has also enhanced anti-tumor immune responses and showed significant inhibition of tumor growth *in vivo*. For instance, Xu et al. developed a mannose-modified liposome-protamine-hyaluronic acid NPs (LPH) for encapsulating with TGF-β siRNA to B16F10 melanoma tumor cells 83. Meanwhile, the authors developed another lipid-calcium-phosphate NP (LCP) to deliver tumor antigens (i.e., Trp 2 peptide and CpG oligonucleotide) to the dendritic cells, with the purpose of eliciting a systemic immune response. The *in vivo* results displayed that silencing of TGF-β by LPH boosted the vaccination efficacy of LCP, and significantly inhibited tumor growth.

The exciting anti-tumor effect produced by the combination of two NPs suggested the combo therapy by two or more therapeutics will induce a more powerful anti-tumor immune response and offer a powerful platform for cancer treatment. Based on this, some strategies that combine RNA-based nanotherapeutics with photodynamic or chemical agents are being reported in recent studies 80, 81. For instance, Dai et al. reported a pH-responsive nanosystem that when co-loaded with PD-L1 siRNA and a mitochondrion-targeting photosensitizer and given to tumor cells, induced the synergistic anti-tumor effect by combing photodynamic and immunotherapy 80. The *in vitro* and *in vivo* results reveal that the nanosystem not only efficiently induced an immune response by photodynamic therapy, but also subsequently induced a siRNA-mediated immune checkpoint blockade that further activated systemic anti-tumor immune responses, and thereby led to significant growth inhibition for melanoma. A similar result has also been reported by Qiao et al. They designed a ROS-responsive nanotheranostic system which combined temozolomide (TMZ)-mediated chemotherapy and immunomodulation by siTGF-β-based therapy on glioblastoma 84. In this study, cationic poly[(2-acryloyl)ethyl(p-boronic acid benzyl)diethyl ammonium bromide] (BA-PDEAEA, BAP) was used to condense with TGF-β siRNA, the zwitterionic lipid-based envelopes (ZLEs) were then coated on polymer-siRNA nanocomplex, and TMZ was loaded into the core of the nanotheranostic NPs (LiB(T+AN@siTGF-β), LBTA). The NPs were finally modified with an angiopep-2 peptide that enabled the NPs to cross the blood-brain barrier (BBB) and accumulate into the glioblastoma tumor region. Both *in vitro* and *in vivo* results demonstrated that this nanotheranostic NPs effectively down-regulated TGF-β expression of tumor cells and dramatically enhanced the efficacy of TMZ mediated chemotherapy. Meanwhile, it showed that the survival time of glioblastoma tumor-bearing mice was significantly prolonged after the synergistic combination treatment.

In addition to directly silencing the immunosuppressive genes, combination therapy with knock out/down oncogenic genes, through siRNA and immune checkpoint blockade therapy, may provide another promising method for cancer treatment. For example, Matsuda et al. used the extracellular vesicles (EVs) to develop a biological nanoparticle-mediated delivery system for intrahepatic delivery of β-catenin siRNA to hepatocellular carcinoma (HCC)^88^. In this study, the β-catenin siRNA EVs and anti-PD-1-based therapy were systemically administrated together. The *in vivo* results demonstrated the therapeutic EVs improved CD8^+^ T cells infiltration and priming, and thus enhanced the anti-tumor effect of anti-PD-1.

#### 3.1.2 Immune cells-targeted siRNA immune-nanotherapy

**Figure 4:**
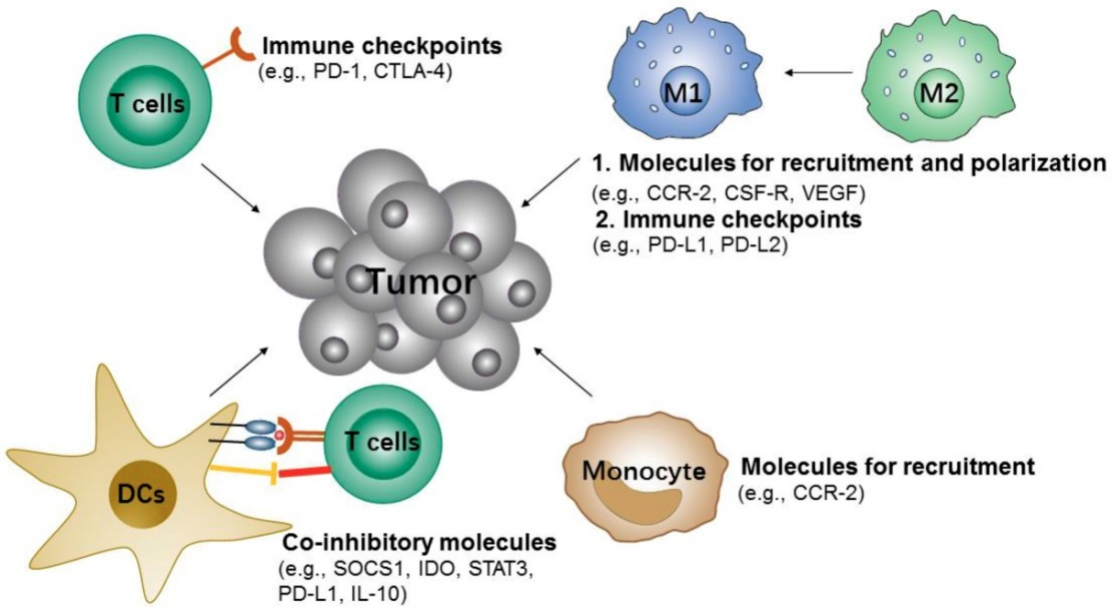
Potential siRNA targets of immune cells for cancer immunotherapy.

During tumor progression, the immune cells can also recognize and eliminate tumor cells, which are known as tumor immunosurveillance. However, tumor cells can also change the host's immune system and escape immune system control by re-education or re-programing the immune cells, e.g., recruitment of various immunosuppressive cells to the primary microenvironment of the tumor, induction of immune cells into pro-tumorigenic types and inhibition of the anti-tumor activity of immune cells 109-111. Therefore, suppressing the immunosuppressive cells or modulating immune cells to anti-tumorigenic types would be an attractive approach to inhibit tumor immune escape and slow tumor growth (Figure 4).

##### 3.1.2.1 T cells

Like cancer cells' highly/over-expressed inhibitory molecules (e.g., PD-L1), some activated T cells also over-express corresponding inhibitory molecules 112, 113, such as programmed cell death protein 1 (PD-1), cytotoxic T-lymphocyte-associated protein 4 (CTLA-4) and mucin-containing protein 3 (TIM-3). All these inhibitory signals are known as immune checkpoints, which can downregulate, T cell activities and therefore prevent the elimination of tumor cells by effector T cells 113. Thus, targeting the immune checkpoints of T cells may also boost the anti-tumor effects. Given this, Li et al. constructed cationic lipid-assisted PEG-PLA-based NPs for efficiently delivering CTLA-4 siRNA to T cells 90. The NPs consisted of poly(ethylene glycol)-block-poly(d,l-lactide) (PEG5k-PLA11k) and the cationic lipid N,N-bis(2-hydroxyethyl)-N-methyl-N-(2-cholesteryoxycarbonyl-aminoethyl) ammonium bromide (BHEM-Chol) polymers which could encapsulate with CTLA-4 siRNA by electrostatic interaction. Although the NPs were only internalized by 4-6% of T cells *in vivo*, it promoted an ~ 2-fold increase in effector CD8^+^ T cells (approximately 40.3% vs. 18.9% of PBS), and the ratio of CD4^+^ FOXP3^+^ Tregs was decreased by ~ 2.5 fold compared to PBS. Accordingly, these NPs could effectively inhibit tumor growth and prolong survival time in mice with melanoma.

##### 3.1.2.2 TAMs

Macrophages are professional phagocytes for eliminating pathogens and cellular debris. In the tumor microenvironment, tumor-associated macrophages (TAMs) usually are either pro-tumor M2 type or anti-tumor M1 type. The macrophages or monocytes are usually recruited to the tumor region and polarized to M1-type at the initial stages of tumor formation, and in advanced tumor progression stage, the macrophages will convert from M1 to M2 type, which thus exerts pro-tumor effects by helping block CD8 T cells 114, 115. Conclusively, reducing the survival and recruitment of TAMs in tumor site or performing targeted delivery of therapeutics to the M2-like TAMs, for depleting them from tumors or converting them to M1 type, would be promising strategies for cancer immunotherapy 116.

Recent studies indicated that the CCL2-CCR2 117 and CCL3-CCR1/CCR5 signaling 118 were required for the recruitment, retention, and the phenotypic recruitment of TAMs. They also indicated that the cytokines 119, including colony stimulating factor 1 (CSF1) and vascular endothelial growth factor (VEGF), are known to recruit monocytes as well. Given this, Qian et al. reported a molecularly-targeted strategy based on dual-targeting nanoparticles (M2NPs) that deliver colony stimulating factor-1 receptor (CSF-1R) siRNA to M2-like TAMs for the specific blockade of the survival of M2-like TAMs, which results in the depletion of them from melanoma tumors 93. The siRNA-carrying M2NPs can inhibit the production of immunosuppressive factors (e.g., IL-10 and TGF-β), but also increase the expression of immunostimulatory cytokines (IFN-γ and IL-12) and CD8^+^ T cells infiltration (2.9-fold). Moreover, it effectively induced the anti-tumor activity of T cells by down-regulating expressions of the exhaustion markers (PD-1 and Tim-3) and stimulating secretion of IFN-γ (6.2-fold). *In vivo* results confirmed that M2NPs led to a dramatic elimination of M2-like TAMs (52%), inhibition of tumor growth (87%), and prolonged survival.

Conde et al. presented peptide-functionalized gold nanoparticles (AuNPs) for delivery of VEGF siRNA to M2-like TAMs where a decrease in the accumulation in lung tumor tissue was observed, alongside enhanced tumor growth inhibition in a lung cancer orthotopic murine model 92. VEGF is a key angiogenic factor, which is highly expressed on M2-like TAMs and well known to promote cancer progression and metastasis. In this study, the authors proved that siRNA mediated gene silencing for inhibiting TAMs accumulation could be achieved by targeting the VEGF pathway, which also indicated that modulation for TAMs would induce an anti-tumor immune response and could be a potential target for cancer treatment.

##### 3.1.2.3 DCs

DCs are the most important antigen-presenting cells (APCs), which could capture, process, and present tumor antigens to either naive CD8^+^ or CD4^+^ T cells. The functions of DCs are either mediating immune tolerance or inducing an anti-tumor immune response. Generally, DCs express a variety of co-inhibitory molecules 120, such as suppressors of cytokine signaling (SOCS) 1, signal transducers and activators of transcription-3 (STAT3), and indoleamine 2,3-dioxygenase (IDO). All of these molecules may suppress the antigen presentation process of DCs. Studies have shown that knockdown of these molecules by RNAi would be an effective strategy for DCs-based immunotherapy 121-124. Based on this, Heo et al. reported a PLGA polymeric NPs that combined the delivery of tumor antigens and SOCS1 siRNA to DCs that induced an enhanced anti-tumor immune response 102. SOCS1 functions as a broadly immuno-suppressive protein which can directly inhibit antigen presentation of DCs to T cells, and it also acts as a negative regulator of Janus kinases (JAKs), which induce immune tolerance. In this study, PLGA polymeric NPs with the loading of SOCS1 siRNA and OVA peptide were efficiently taken up by bone-marrow-derived dendritic cells (BMDCs) and this showed proof of the significant knockdown effect on the expression of SOCS1. The downregulation of SOCS1 in BMDC further led to a drastic enhancement in cytokine production, and finally resulted in the essential induction of anti-tumor immune response.

##### 3.1.2.4 Others

Neutrophils has been described as part of the innate immune response, but recent studies have shown that neutrophils can be a key negative regulator for adaptive immune responses by suppressing T cell proliferation, and therefore they also be so-called myeloid-derived suppressor cells (MDSCs)^125^. MDSCs have been identified to facilitate the development of an immunosuppressive tumor microenvironment 126. Many studies have also demonstrated MDSCs are critically involved in immunosuppressive effects through the high expression of metabolic enzymes 127, 128, such as IDO, and arginase-1 (ARG1), cytokines^129^, ^130^, and chemokines, such as TGF-β and IL-10. Meanwhile, the recruitment of MDSCs relies on the chemokines 131 (e.g., CCL2). Due to the important role in tumor-associated immune suppression of MDSC, many studies were focused on exploring therapeutic strategies to eliminate these cells or to modulate their functions 132-135. For example, Leuschner et al. introduced monocyte-targeting lipid NPs for the delivery of CCR2 siRNA to the inflammatory monocyte 104. The results showed efficient inhibition of CCR2 expression by siRNA-mediated CCR2 gene silencing in monocytes that prohibited their accumulation in inflammatory sites and reduced the number of TAMs.

### 3.2 Nanostructure-mediated microRNA delivery for immunotherapy

Non-coding RNAs are usually classified into small non-coding RNA (e.g., microRNA) and long non-coding RNA (lncRNA, > 200 nt), depending on their size. Recent studies of lncRNAs have indicated that lncRNAs function as crucial regulators in various immunity processes, including immune cells differentiation and function, regulation for tumor microenvironment, but the detail regulatory mechanisms remain unclear 136. While current studies are mainly focused on the development of the lncRNAs for immune regulation and the functional relationship between lncRNAs and immunity, and there were limited reports on lncRNAs-based nanomaterials for immunotherapy, we might believe that lncRNAs could become a novel therapeutic agent in cancer immunotherapy.

In addition, recently studies have shown that microRNAs are associated with the modulation of various pathways of cancer and immune cells. MicroRNAs usually produce competition for oncogenic and tumor suppressive effects by blocking either tumor suppressive mRNA or oncogenic mRNA 137-139. Generally, oncogenic microRNAs are highly/over-expressed in cancer cells, while tumor-suppressive microRNAs are under-expressed. Restoration of tumor-suppressive microRNAs could both inhibit cancer cell proliferation and induce cell apoptosis. Because of these possibilities, this technique has been viewed as novel therapeutics for cancer treatment 6, 140. Ultimately, it is necessary to carefully assess the specific roles of microRNAs in cancer or immune cells, and develop an effective and safe microRNA-based therapeutic for cancer treatment.

#### 3.2.1 Tumor cells-targeted microRNA immunonanotherapy

As mentioned above, tumor cells will up-regulate the expression of immune checkpoint proteins, or “don't eat me” signals, to construct an immunosuppressive microenvironment. microRNA acts as a gene regulator, which can either be used to directly inhibit expressions of immune escape factors and pro-inflammatory cytokines or act as oncogenic factors for facilitating immune system evasion by tumor cells^141^. For example, many microRNAs including miR-34a, miR-200, miR-142-5p and miR-424, et al. have been found to be involved in PD-L1 expression levels in several cancer cells 142, 143. Among them, miR-424 not only activates T cells immune response through direct targeting of PD-L1, but also can restore the chemosensitivity of ovarian cancer cells 144. However, some microRNAs, such as miR-20b, miR21, and miR-130b, have been found to inhibit PTEN expression in colorectal cancer, but they in turn promote PD-L1 upregulation 145. MicroRNA antagonists are short, single-stranded oligonucleotide molecules complementary to microRNA sequences that have recently been used for targeting and reducing microRNA activity. Therefore, using antagonists of microRNA will also decrease oncogenic microRNA activity and provide promising results of targeted genes for immunomodulation. To date, there has been a limited report by using nanotechnology for the delivery of microRNA to tumor cells for triggering an anti-tumor immune response, but there is high confidence that it would be a potential strategy for cancer immunotherapy.

#### 3.2.2 Immune cells-targeted microRNA immunonanotherapy

**Figure 5:**
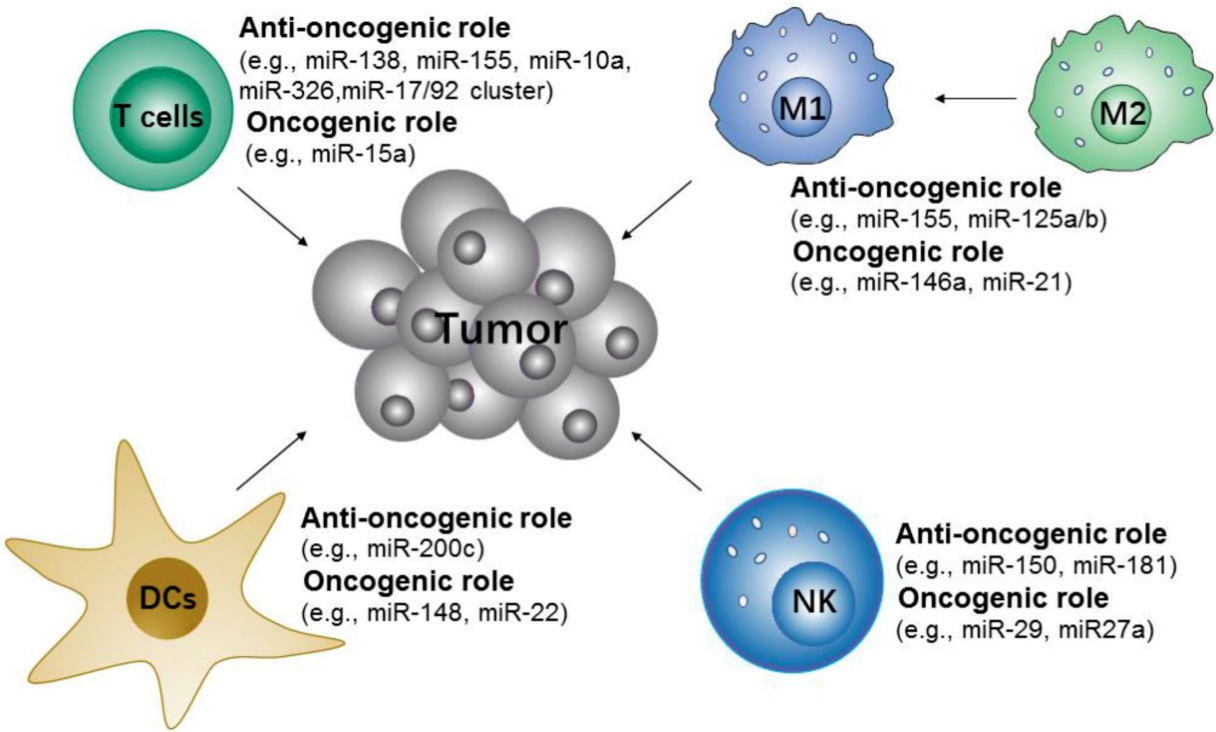
Potential microRNA targets of immune cells for cancer immunotherapy. The microRNAs either contribute to or repress the immune cells to initiate anti-tumor responses.

Many studies have shown that microRNAs are important in the modulation of innate and adaptive immune-responses via controlling the differentiation and activation of immune cells and the maintenance of immune proinflammation factors 141. These microRNAs either contribute to or repress the immune cells to initiate anti-tumor responses (Figure 5). Specifically, the upregulation of miR-146a 146 and miR-21^147^ attenuate M1-like TAMs activation by targeting TLR/NF-κB pathway, but miR-155^148^, ^149^ promote M1-like TAMs polarization by targeting SOCS1 or targeting the negative regulator of NF-κB and TNF-α-induced protein 3. Along with TAMs, some microRNAs, such as miR-150 and miR-181a/b, have been found to control the differentiation of NK cells 150. The use of miR-148 and miR-22 inhibitors may also promote DCs maturation 151-153. In addition, the differentiation and functions of different T cell subsets are regulated by microRNAs 154, 155. For example, miR-155 are more in favor of Th1 phenotype 156, miR-326 promote Th17 differentiation 157, and miR-10a and miR-17/92 cluster regulate T follicular helper maturation 158.

Given the potential applications of microRNA-based therapeutics in cancer immunotherapy, a variety of microRNA-based nanomaterials that can efficiently target immune cells for immunomodulation have been developed (Table 3). For instance, Zhang et al. designed lipid-coated calcium phosphonate nanoparticles (CaP/miR@MNPs) which were further conjugated with mannose for specific delivery of miR155 to TAMs 159. The results demonstrated that CaP/miR@MNPs could successfully transfer pro-tumor M2-like TAMs to antitumor M1-like TAMs, and therefore elicit a potent antitumor immune response, whilst inhibiting tumor growth. Similarly, Parayath et al. developed a CD44 targeting hyaluronic acid-poly(ethylenimine) (HA-PEI)-based nanoparticle for the delivery of miR-125b to peritoneal macrophages 160. Overexpression of miR-125b in macrophages would promote M1-like TAMs activation and lead to inhibition of primary tumor growth and metastasis. *In vivo* results showed that there was a more than 6-fold increase in the ratio of M1 to M2 TAMs and 300-fold increase in the ratio of iNOS (M1 marker) to Arg-1 (M2 marker) in TAMs after treatment with HA-PEI-125b nanoparticles. All of the examples above indicated that successfully inducing M1-like TAMs polarization would enhance anti-cancer immunotherapy.

### 3.3 Nanostructure-mediated mRNA delivery for immunotherapy

mRNA emerging as a cancer therapeutic has drawn increasing attention due to its multiple unique features^166^, such as the absent risk of insertional mutagenesis, more consistent and predictable kinetics of protein expression, and relatively convenient *in vitro* synthesis. However, its poor stability (easily degraded by the nucleases) and propensity for immunostimulation have greatly hindered the *in vivo* application. Moreover, it very difficult for the larger mRNA molecules with negative charges to enter antigen-presenting cells (APCs) directly. Nanoparticle-based platforms with high cytosolic transportation and reduced renal filtration, could be emerging as a promising mRNA delivery tool to protect nucleic acids from enzymatic degradation and withstand multiple intracellular and extracellular barriers 35, 167. Here, we will describe two examples to present the advances of mRNA nanomedicines in immunotherapy applications, including vaccination and cell engineering.

#### 3.3.1 mRNA-based NPs for vaccination

**Figure 6:**
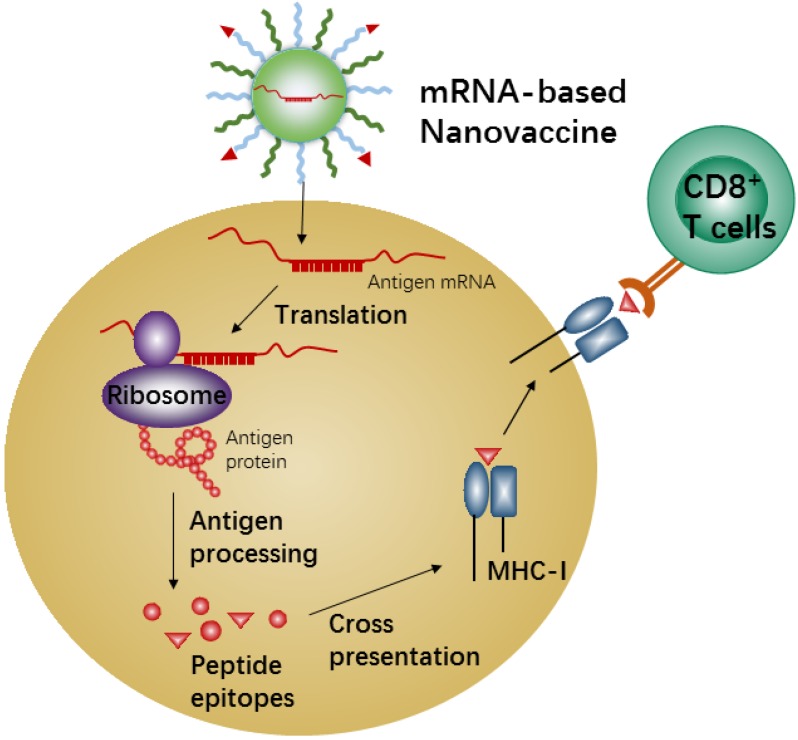
Schematic illustration of antigen cross-presentation by mRNA-based nanovaccine in APC.

mRNA-based vaccines show a promising alternative due to the high potency, safe administration, and capacity for rapid development and low-cost manufacture^168^, ^169^. Recent nanotechnological advances have largely overcome the issues of *in vivo* mRNA delivery, and therefore mRNA-based nanovaccines have recently attracted increasing attention because of the promising results achieved in many anti-tumor vaccination studies in animal models 170, 171. Meanwhile, the mRNA-based vaccine could effectively carry out antigen-encoding mRNA to antigen presenting cells (APCs) *in vivo* directly. Also, the mRNA-based vaccine is unlimited in molecular structures and number of tumor antigen proteins. When the nanocarriers efficiently deliver these antigen-encoding mRNAs into APCs, the mRNAs will be released and translated into tumor antigenic proteins in the cytoplasm of APCs, which are then processed into peptide epitopes for subsequent binding with the major histocompatibility complex (MHC) class I *via* cross-presentation pathway. The MHC-peptides are finally transferred to the cell surface of APCs for activation of CD8^+^ T cells, leading to corresponding anti-tumor immune responses (Figure 6). Efficient *in vivo* mRNA delivery and induction of strong cytotoxic T cell response are the key prerequisites for cancer immunotherapy by mRNA-based nanovaccines. There are so many advantages and disadvantages for *in vivo* application of mRNA-based nanovaccines, and many review articles have summarized the advertences of mRNA-based nanovaccines in cancer therapy. Here we just describe one example to present the mechanism of anti-tumor response by nanovaccine.

A lipid nanovaccine with the loading of tumor-associated antigens mRNA (e.g., gp100 and TRP2) was designed by Oberli et al. 172. In this study, the lipid nanoparticle that consisted of an ionizable lipid, a lipid-PEG, a phospholipid, cholesterol, and an additive was developed. The ionizable lipid was used for complexation with the negatively charged mRNA and also helped with cellular uptake. The results showed the NPs worked well for the delivery of mRNA to dendritic cells, macrophages, and neutrophils, and effectively led to strong activation of CD8^+^ T cells after a single immunization in the B16F10 melanoma model. Moreover, after treatment with these NPs, B16F10 melanoma tumor experienced shrinkage, and mice survival was significantly extended. The exciting result from this study demonstrated that the induction of cytotoxic T cell response by mRNA nanovaccine would be an excellent candidate in the application of cancer treatment.

In the nanovaccine field, the nanostructures with CpG oligodeoxynucleotides that act as immuno-adjuvants for inducing immunostimulatory responses are very attractive. CpG oligodeoxynucleotides are Toll-like Receptor 9 (TLR9) agonists, which are expressed in human B and plasmacytoid dendritic cells. Recent studies demonstrated CpG not only could induce apoptosis of tumor cells with high-expression of the TLR9 receptor, but also enhance NK cell activation and promote activation of anti-tumor immune response by combining with various of cytokine treatments 173, 174. Therefore, CpG-based nanotherapeutics can only benefit from combination with strategies of immune checkpoint blockade.

### 3.3.2 mRNA-based NPs for T cell engineering

As a novel immunotherapy method, CAR-T therapy has achieved great success in treating patients with hematological malignancies 13, such as leukemia and lymphoma therapy. Currently, the most common techniques for the development of CAR-engineered T cells are using viral gene transduction by virus-mediated delivery. However, using viral vectors may lead to the potential insertional mutagenesis and genotoxicity for effector T cells^15^. Meanwhile, the feared side-effects would happen when virus vectors transduced cells 175. Hence, more precise T cell manipulations are currently under investigation.

**Figure 7:**
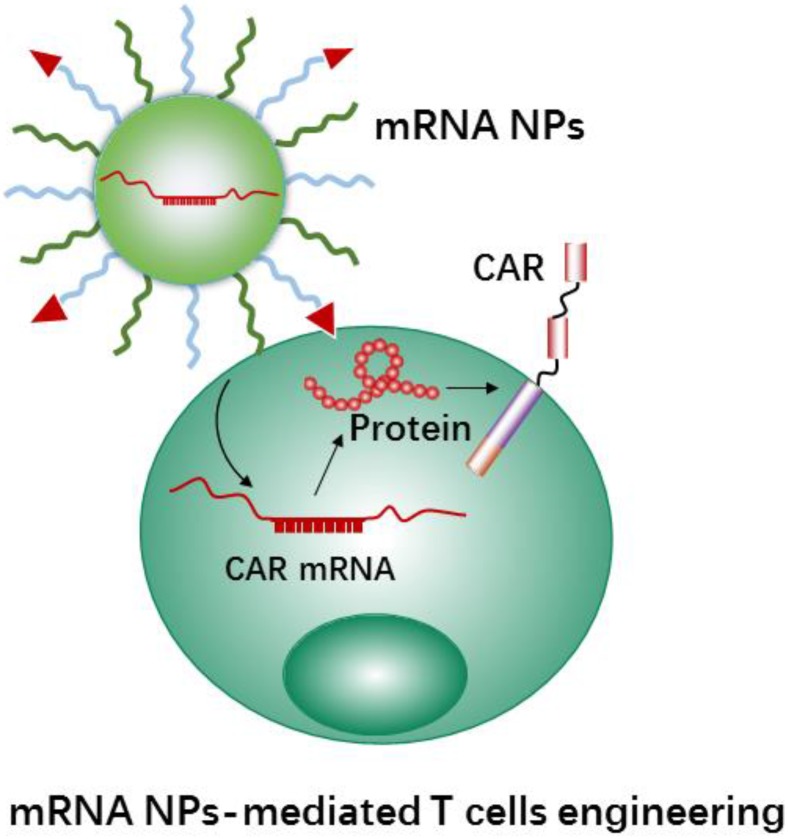
Schematic representation of mRNA nanotherapeutics for T cell engineering. The nanocarriers delivery CAR mRNA to T cells, and induce T cells activation by expressing the CAR protein on the surface of T cells.

Using mRNA instead of DNA as T cell engineering therapeutics (Figure 7) is attractive due to the multiple unique features of mRNA 176, 177. Meanwhile, the use of nanocarriers protect mRNA therapeutics from degradation and may help with cellular uptake and endosomal escape. In addition, nanocarriers provide further advantages for the engineering of immune cells, e.g., coating with specific ligands for increasing cell binding and cellular uptake, and carrying multiple RNA payloads. Moffett et al. developed a targeted nanocarrier for reprogramming T cells by delivery of several mRNAs to T cells 178. The nanocarrier consisted of negatively charged polyglutamic acid (PGA), poly(β-amino ester) (PBAE) polymers and T cell targeting ligands (e.g., anti-CD3 and anti-CD8). In this study, the authors first used their nanocarrier to deliver FoxO1 (Forkhead box O1, a transcription factor to promote the generation and maintenance of memory T cells) mRNA to CAR T cells for overexpressing FoxO1. FoxO1 overexpression can bias CAR-T-cells toward a central memory phenotype and therefore improve the anti-tumor activity of CAR T cells. In addition, the authors developed mRNA nanocarriers to transiently express genome-editing proteins (CRISPR) for efficiently knocking out T cells receptors in CAR-programmed lymphocytes. The results demonstrated that the NPs could effectively transport mRNAs to targeted T cells, and subsequently led to the targeted T cells to express selected proteins. Next, we will describe CRISPR-Cas9 nanotechnology mediated cell engineering and its application in immunotherapy.

### 3.4 CRISPR-Cas9 nanotechnology for cell engineering

**Figure 8:**
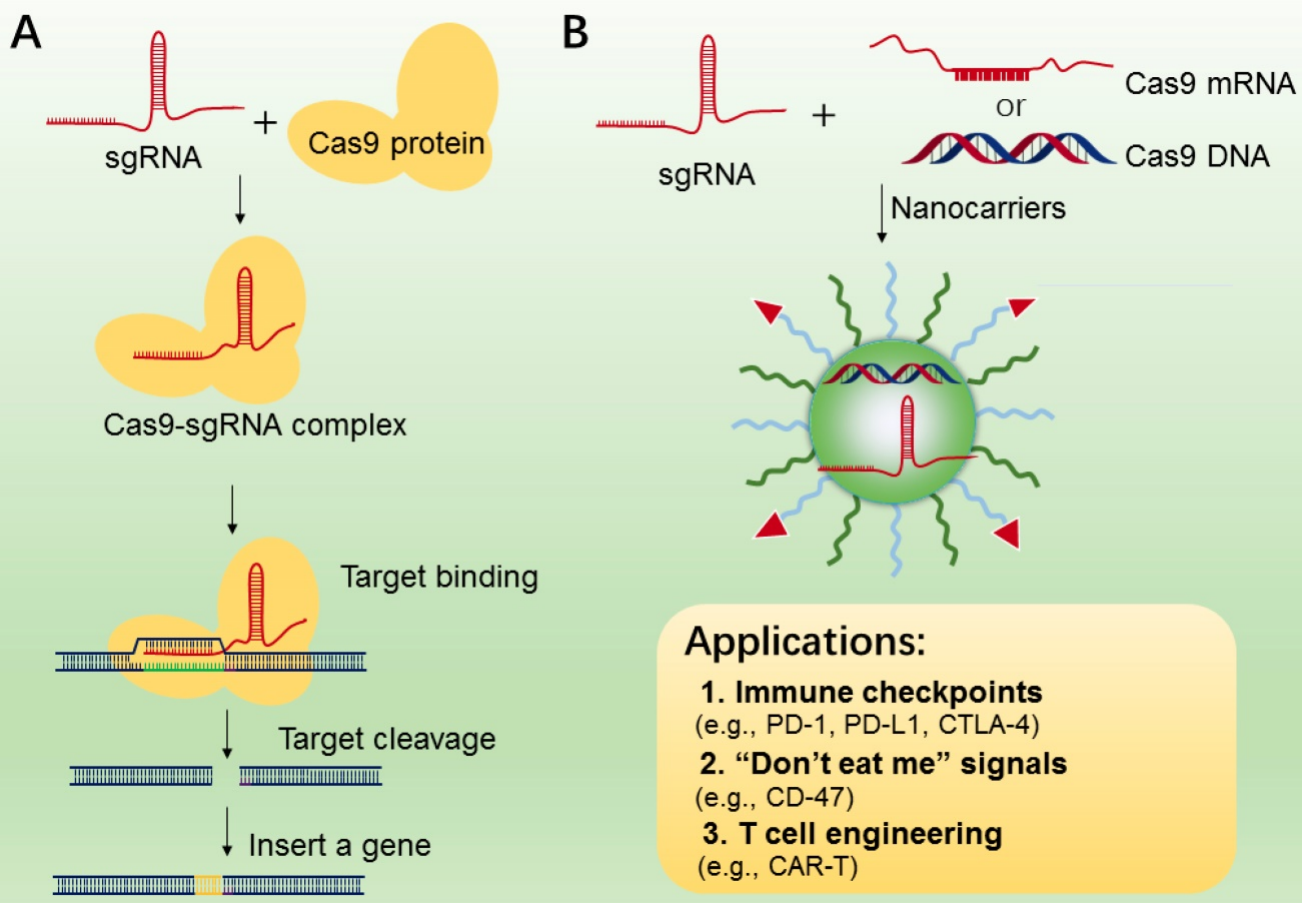
A. The mechanism of CRISPR-Cas9 technology for gene editing. B. CRISPR-Cas 9 nanotherapeutics for cell engineering and the applications in cancer immunotherapy. The CRISPR-Cas 9 system (sgRNA with Cas9 mRNA or DNA or protein) was transported to tumor or immune cells by nanocarriers.

CRISPR/Cas9 system (clustered regularly interspaced short palindromic repeats) 178, is an RNA-guided DNA targeting technology, which has been widely applied to genome editing 180, 181, gene therapy 182 and manipulation of induced pluripotent stem cells (iPS) studies^183^. The CRISPR-Cas9 gene editing machinery is comprised of two essential components, i.e., sgRNA and DNA endonuclease Cas9 (Figure 8A). The Cas9 protein functions in locating and cleaving targeted DNA, and the guide RNA has a 5′ end that is complementary to the target DNA sequence. Only when two macromolecules are forming a complex, will the cleavage activity of Cas 9 begin to be triggered (Figure 8A). In cancer immunotherapy, CRISPR-Cas9-mediated genome editing is usually applied to knockout the genes that encode inhibitory receptor proteins of tumor or T cells, such as PD-1, PD-L1, and CTLA-4^184^-^186^. In 2016, a case of CRISPR-Cas9 application in a clinical trial of T cells engineering by deleting PD-1 was performed in China, and the result was promising^187^, indicating the potential value of CRISPR-Cas9-mediated genome editing in immunotherapy.

While CRISPR-Cas9 has been used as a particularly versatile and operationally simple tool for gene editing in many cell types, the effective delivery of CRISPR-Cas9 system into targeted T cells is still the common issue for efficient genome editing. In addition, off-target effects are also found in many CRISPR-Cas9-mediated gene-editing studies 188. To reduce these effects, various nanocarriers have been developed as the delivery of CRISPR-Cas9 system^189^-^191^, which may be applied to manipulations of immune cells (TAMs, B cells, and T cells) and tumor cells (Figure 8B). In addition, compared to the viral vectors with high immunogenicity, various types of lipid-based or polymer-based nanocarriers, which are actively targetable with low immunogenicity, showed exciting delivery efficiency^192^, ^193^. For example, Ray et al reported a nanomaterial platform based cationic arginine-coated gold nanoparticles^194^, which delivered CRISPR-Cas9 gene editing machinery into macrophage cells for knockout expression of “don't eat me” signals (SIRP-α). The NPs based nanoplatform has shown ∼90% delivery efficiency as well as ∼30% gene editing efficiency. The *in vitro* experimental results also showed a 4-fold increase in the innate phagocytic capabilities of the macrophages by using this strategy to turn off the “don't eat me” signal on macrophages, indicating this strategy may be a promising tool for the development of “weaponized” TAMs for cancer immunotherapy. Similarly, Cheng et al. proposed a double emulsion method by complexing plasmids with stearyl polyethyleneimine (stPEI) as the core to form human serum albumin (HSA) (plasmid/stPEI/HSA) NPs for delivery of CRISPR/Cas9 195. The NPs could disrupt or silence the expression of PD-L1 by CRISPR/Cas9-mediated gene editing.

## 4. Conclusions and future perspectives

During the past few years, immunotherapy has shown to be one of the most promising therapeutic strategies and has resulted in a paradigm shift in the treatment of cancers 13, 112. RNA-based therapeutics provide several advantages in immunomodulation and cancer vaccines, which will serve as a competitor and as a current immunotherapies partnership to achieve synergistic combinations for more efficient and personalized results 75, 169, 176, 196. Compared to the conventional proteins, antibodies, and cell-based therapeutics (e.g., CAR-T), RNA-based therapeutics including siRNA, microRNA, and mRNA can either knock out or knock down targeted genes or upregulate expressions of specific proteins, and such features of RNA-based therapeutics cause high selectivity and low risk of off-target hitting. Meanwhile, RNA-based therapeutics have become much more diverse and broad in regulatory functions in immunotherapy than the other antibody or protein-based therapies. For example, RNA-based therapeutics could activate rapid and effective anti-cancer immune response by inducing immunogenic cell death of tumor cells, and thus may skip the requirement of deep tumor penetration-a significant hurdle faced by traditional cancer nanomedicines. Moreover, the development and production of RNA therapeutics are very convenient, rapid and cost-effective 197. The success of pre-clinical studies has led to the initiation of clinical trials of different forms of RNA therapeutics for cancer immunotherapy (Table 4).

Given the potentials of these RNA therapeutics, various nanoparticle-based delivery systems, such as lipid-based, polymeric, inorganic and bio-inspired nanomaterials have been explored extensively 49, 167, 198, 199. These nanoparticle-based delivery systems not only carry a high dose of therapeutic payloads to targeted cells or tissues, but also show the same regulatory functions in immunotherapy with RNA therapeutics. In addition, the combination of RNA-mediated nano-immunotherapy with chem- or photodynamic therapies demonstrated promising results in an animal model^81^, ^106^. Meanwhile, combining RNA-mediated nanotherapy with current immunotherapies (e.g., anti-CTLA-4, anti-PD-1, and anti-PD-L1) is also a great opportunity to enhance cancer treatments 133. However, there are still several issues need to be solved before such applications are translated to the clinic.

To begin with, some of the main barriers that hinder the stability and *in vivo* safety of the various nanomaterial carriers include cytotoxicity and undesirable immune stimulation 21, 200. As mentioned previously, potential *in vivo* toxicity issues can arise from the use of cationic units in polymeric NPs, payload leakages from lipid-based nanostructures, and the non-biodegradability of inorganic NPs 20, 38. In addition, the NPs may be recognized as a foreign substance, and thus easily excreted by renal/hepatic clearance or eliminated by the innate immune systems 198. To address these issues, nanocarriers should be bio-compatible and capable of biodegradability by modulating the surface of the NPs with biomolecules or proper hydrophilic materials such as PEG.

Secondly, nanocarriers must increase cell targeting and cell internalization. For example, for targeting tumor cells to silence PD-L1 by RNA-based NPs, it needs to target as many of them as possible, thus requiring high accumulation and deep tumor penetration. To increase cellular uptake and thus increase the effectiveness of RNA delivery, NPs could be coated with targeting ligands, which would increase the chance of binding to the targeted cells. In addition, effective on-demand RNA delivery and release would become feasible through the incorporation of backbones of that are sensitive to different stimuli, either endogenous (e.g., pH, enzyme, and redox) or exogenous (e.g., temperature, electricity, light, magnetic force, ultrasound)^201^, ^202^. Such a feature of nanocarriers would enable optimal spatial and temporal release of RNA payloads. Despite many nanovesicles infiltrating the targeted cells through the endocytic pathway, they must still overcome the barriers that endosomes and lysosomes pose before achieving the ultimate goal of cytosolic release of RNA therapeutics 203. Generally, nanomaterials are transported into the endosome, which then fuses with lysosomes and ultimately destroys the RNA cargoes. Some specific targeting peptides (e.g., HA) or surfactants can be used in the preparation of nanocarriers to enable the endosomal/lysosomal escape via the proton sponge effect or membrane lysis. It should be noted that many stimuli-responsive nanomaterials had potential dose-dependent cytotoxicity. This cytotoxicity was due to the stimuli-responsive motif often being limited by insufficient biocompatibility or the need for bio-degradability 204. Meanwhile, the complexity of the architectural design and difficulties in the synthesis of these NPs are likely to hamper their clinical translation. Therefore, the benefit-to-risk ratio for the development of responsive NPs needs to be balanced, and the issues related to the responsive characteristics would eventually need to be solved.

In summary, the goal of the current review article was to summarize and highlight the RNA-based nanotherapeutics for the immunomodulation and enhancement of cancer immunotherapy. Given the convenience and importance of RNA therapeutics in cancer immunotherapy, using nanomaterials for effectively delivery of RNA to a targeted tumor or immune cells for triggering anti-tumor immune response have already produced some exciting results in the treatment of cancer. Meanwhile, by combining RNA-mediated immunomodulation with ICB immunotherapy, chemotherapy, and photodynamic therapy, synthesized effects have been noted and a bright future for the clinical use of cancer treatment awaits. Although the RNA delivery nanoplatforms for clinical applications is still challenging, some issues need to be solved before this nanotherapeutics translated from the bench to the bedside. We believe that the RNA-mediated nano-immunotherapy has great potential to overcome some of these shortcomings and has the opportunity to be used for cancer treatment in the future.

## Figures and Tables

**Table 1:** Nanoparticle-based platforms for RNA delivery

Nanocarriers	Classifications	Advantages	Disadvantages
Lipid-based nanostructures	Liposomes; solid lipid nanoparticles; lipid emulsions	Easy preparation, good biocompatibility and biodegradability	Limited stability, easy leakage of payloads, and rapid clearance
Polymer-based nanomaterials	Natural or naturally derived polymers: chitosan, poly-l-lysine, atelocollagen, etc. Synthetic polymers: PLGA, PEI, PVA, PLA, PEG, etc.	Good biocompatibility and biodegradability for natural or naturally derived polymers, low cost of production, stimulation of drug release, easy modification	Nondegradable for some responsive polymers, dose-dependent toxicity
Inorganic NPs	MSNs, CNTs, QDs, and metal nanoparticles (e.g., iron oxide and gold nanoparticles)	Easy surface modification, good reproducibility, and easy cell uptake	Non-biodegradability, potential toxicity
Bio-inspired nano-vehicles	DNA-based nanostructures, exosome-mimetic nanovesicles, red blood cell member-based ghosts	Good biodegradability, low toxicity, strong targeting and low immune induction	High cost, stability concern

**Table 2:** Summary of siRNA-based nanotherapeutics for tumor immunotherapy

Cells	Nanocarriers	Targeted gene	Immunological effects	Ref.
Tumor cells	PCL-PEG/PCL-PEI	PD-L1	Checkpoint blockade	78
	FA-PEI polymers	PD-L1	Checkpoint blockade	79
	Acidity-Responsive Micelleplex	PD-L1	Checkpoint blockade	80
	Acid-activatable micelleplex (PDPA based)	PD-L1	Checkpoint blockade	81
	Au-CGKRK nanoconjugates	PD-L1, STAT3	Anti-proliferation and checkpoint blockade	82
	Liposome-protamine-hyaluronic acid NPs	TGF-β	Decrease TGF-β and enhance the antigen-specific immune response	83
	ROS-responsive NPs	TGF-β	Modify the immunosuppress microenvironment	84
	HA-coated liposome	CD47	Decrease immune escape of tumor cells	85
	Glutamine-functionalized branched polyethyleneimine	CD47	Induce evasion of phagocytic clearance	86
	Chitosan lactate	CD-73	Attenuate the immunosuppressive microenvironment of the tumor	87
	The extracellular vesicles (EVs)	β-catenin	Combo therapy with ICB	88
T cells	Lipid-coated calcium phosphate (LCP)	PD-1	Checkpoint blockade	89
	PEG-PLA	CTLA-4	Checkpoint blockade	90
TAMs	Gold NPs	TNF-α	Silence pro-inflammatory cytokines	91
	Gold NPs	VEGF	Reduce the recruitment of inflammatory TAMs	92
	Peptides NPs	CSF-1R	Elimination of M2-like TAMs	93
DCs	Gold nanorods or GNRs-PEI	IDO	Promote DCs maturation, and increase secretion of pro-inflammatory cytokines	94, 95
	Cationic lipid NPs	PD-L1, PD-L2	Checkpoint blockade	96
	PEI based NPs	PD-L1	Induce immunosuppressive DCs to antigen-presenting cells	97
	PEI based NPs	IDO	Increase secretion of proinflammatory cytokines	98
	PEG-PLL-PLLeu polypeptide micelles	STAT3	Induce DCs maturation and activation, elevate expressions of CD86 and CD40 and IL-12 production	99
	PLGA NPs	STAT3	Induce DCs maturation and promote antigen cross-presentation	100
	Cationic lipid NPs	SOCS1	Promote production and release of pro-inflammatory cytokines	101
	PLGA NPs	SOCS1	Enhance the production and release of pro-inflammatory cytokines	102
	lipid envelope-type NPs	A20	Enhance production of pro-inflammatory molecules after lipopolysaccharide stimulation	103
Others	Lipid/PEG NPs	CCR2	Prevent monocytes accumulation	104
	PEG/MT/PC NPs	VEGF, PIGF	Anti-proliferation and reverse immune environment	105
	Chitosan NPs	Galectin-1	Reduce polarization to M2 TAMs	106

**Table 3:** Summary of microRNA-based nanotherapeutics for cancer immunotherapy

Cells	Nanocarriers	microRNA	Immunological effects	Ref.
T cells	Exosome-like nanovesicles	miR-150 Antagonist	T-cell regulation	160
TAMs	Layered double hydroxides NPs	miR-155	Repolarize M2 to M1	161
	Lipid-coated NPs	miR-155	Repolarize M2 to M1	158
	CD44 coated HA-PEI based NPs	micR-125b	Reprogram TAMs into M1	159
DCs	Exosomes	miR-155	Increase the expressions of MHC-II, CD86, CD40, and CD83, and promote the secretion of the IL12p70, IFN-gamma, and IL-10	162
	PEG-PLL-PLLeu polymeric NPs	miR-148a Antagonist	Reprogram DCs, reduce Treg cells and myeloid-derived suppressor cells	163
NK cells	Exosomes	miR-186	Promote NK activation	164

**Table 4:** Current clinical studies of RNA-mediated immunotherapy for the treatment of cancer

Targeting Cell	RNAs Encoding	Cancer Types	Status	ClinicalTrials.gov Identifier Number
T cells	MET scFv CAR	Malignant Melanoma, Breast Cancer	Early Phase 1 Recruiting	NCT03060356
	cMet CAR	Metastatic Breast Cancer; Triple Negative Breast Cancer	Phase 1 Completed	NCT01837602
	Chimeric anti-mesothelin immunoreceptor SS1	Pancreatic Cancer	Phase 1 Completed	NCT01897415
DCs	TAAs: NY-ESO-1, MAGEC1, MAGEC2, 5 T4, Survivin, and MUC1	Lung Cancer	Phase 2 Recruiting	NCT03164772
	TAAs: PSA, PSCA, PSMA, STEAP1, PAP and MUC1	Prostate Carcinoma	Phase 2 Completed	NCT02140138
	Neo-Ag	Melanoma	Active No Recruiting	NCT02035956
	Neo-Ag	Solid tumor	Phase 1 Recruiting	NCT03313778
	Neo-Ag	Melanoma; Colon Cancer; Gastrointestinal Cancer; Genitourinary Cancer; Hepatocellular Cancer	Phase 2 Completed	NCT03480152
	Three variant RNAs; p53, and Neo-Ag based on NGS screening	Breast Cancer (Triple Negative Breast Cancer)	Phase 1 Recruiting	NCT02316457
	Carcinoembryonic antigen RNA	Colorectal Cancer; Metastatic Cancer	Phase 2 Completed	NCT00003433
	Prostate specific antigen (PSA)	Prostate Cancer	Phase 2 Completed	NCT00004211
	Carcinoembryonic antigen	Breast Cancer; Colorectal Cancer; Extrahepatic Bile Duct Cancer	Phase 1 Completed	NCT00004604
	Total tumor RNA	Kidney Cancer	Phase 1 Completed	NCT00005816
	Autologous tumor RNA	Melanoma	Phase 3 Recruiting	NCT01983748
	TAAs: NYESO-1, MAGE-A3, tyrosinase, and TPTE	Melanoma	Phase 1 Recruiting	NCT02410733
	siRNA: LMP2, LMP7, and MECL1; mRNA: MART-1, tyrosinase, gp100, and MAGE-3	Melanoma	Phase 1 Completed	NCT00672542
	Melan-A, Mage-A1, Mage-A3, Survivin, GP100 and Tyrosinase	Malignant Melanoma	Phase 1/2 Completed	NCT00204516
	pp65-flLAMP	Glioblastoma	Active No recruiting	NCT03615404

## Abbreviations

siRNA: small interfering RNA
mRNA: messenger RNA
ICB: immune checkpoint blockade
CAR-T: chimeric antigen receptor T-cell immunotherapy
RISC: RNA-induced silencing complex
AGO2: Argonaute-2
pri-microRNA: primary microRNA
pre-microRNA: precursor microRNA
UTR: untranslated region
ORF: open reading frame
NPs: nanoparticles
EPR: enhanced permeability and retention effect
PLGA: poly(lactic-co-glycolic acid)
PEI: polyethyleneimine
PEG: polyethylene glycol
PVA: polyvinyl alcohol
PLA: poly-l-lactic acid
MSNs: mesoporous silica nanoparticles
CNTs: carbon nanotubes
QDs: quantum dots
PCL-PEG: polycaprolactone-polyethyleneglycol
PCL-PEI: polycaprolactone-polyethylenimine
FA-PEI: folic acid- polyethylenimine
PDPA: poly(2-(diisopropylamino)ethyl methacrylate
PEG-PLL-PLLeu: poly(ethylene glycol)-b-poly(l-lysine)-b-poly(l-leucine)
PEG /MT/PC NPs: polyethylene glycol (PEG)/mannose doubly modified trimethyl chitosan ( MT)/poly (allylamine hydrochloride) (PC)-based nanoparticles (NPs)
HA: hyaluronic acid
AuNPs: gold nanoparticles
OVA: Ovalbumin
TME: tumor microenvironment
HCC: hepatocellular carcinoma cells
TGF-β: transforming growth factor-β
VEGF: vascular endothelial growth factor
IL-6: interleukin-6
CXCL5: C-X-C motif chemokine 5
PD-1: programmed death receptor-1
PD-L1: programmed death receptor-1 ligand
CTLA-4: cytotoxic T-lymphocyte-associated protein 4
TIM-3: mucin-containing protein 3
CSF1: colony stimulating factor 1
CSF-1R: colony stimulating factor-1 receptor
IFN-γ: interferon γ
TNF-α: tumor necrosis factor α
Tregs: regulatory T cells
Th cells: T helper cells
DCs: dendritic cells
TAMs: Tumor-Associated Macrophages
NK: natural killer cells
MDSCs: myeloid derived suppressor cells
APCs: antigen-presenting cells
MHC: major histocompatibility complex
SOCS1: suppressor of cytokine signaling 1
IDO: indoleamine 2,3-dioxygenase
STAT3: signal transducer and activator of transcription-3
ARG1: arginase-1
TLR: Toll-like receptors
NOS: nitric oxide synthase
FoxO1: Forkhead box O1
CRISPR: clustered regularly interspaced short palindromic repeat
MHC: major histocompatibility complex
